# Plant-based dietary changes may improve symptoms in patients with systemic lupus erythematosus

**DOI:** 10.1177/09612033211063795

**Published:** 2022-01-03

**Authors:** Aziyadé Knippenberg, George A Robinson, Chris Wincup, Coziana Ciurtin, Elizabeth C Jury, Anastasia Z Kalea

**Affiliations:** 1 Division of Medicine, 4919 University College London, London, UK; 2 Centre for Rheumatology Research, Division of Medicine, 4919 University College London, London, UK; 3 Centre for Adolescent Rheumatology Versus Arthritis, Division of Medicine, 308348 University College London, London, UK; 4 Institute of Cardiovascular Science, 4919 University College London, London, UK

**Keywords:** Lupus, SLE, diet, dietary patterns, plant-based, symptoms, autoimmune

## Abstract

**Introduction:**

Previous studies have reported that patients affected by systemic lupus erythematosus (SLE) are interested in using diet to treat fatigue, cardiovascular disease and other symptoms. However, to date, there is insufficient information regarding the ways for patients to modify their diet to improve SLE symptoms. We investigated the relationship between the eating patterns of SLE patients and their self-reported disease symptoms and general aspects of health.

**Methods:**

A UK-based, online survey was developed, in which patients with SLE were asked about their attitudes and experiences regarding their SLE symptoms and diet.

**Results:**

The majority (>80%) of respondents that undertook new eating patterns with increased vegetable intake and/or decreased intake of processed food, sugar, gluten, dairy and carbohydrates reported benefiting from their dietary change. Symptom severity ratings after these dietary changes were significantly lower than before (21.3% decrease, p<0.0001). The greatest decreases in symptom severity were provided by low/no dairy (27.1% decrease), low/no processed foods (26.6% decrease) and vegan (26% decrease) eating patterns (p<0.0001). Weight loss, fatigue, joint/muscle pain and mood were the most cited symptoms that improved with dietary change.

**Conclusion:**

SLE patients who changed their eating patterns to incorporate more plant-based foods while limiting processed foods and animal products reported improvements in their disease symptoms. Thus, our findings show promises in using nutrition interventions for the management of SLE symptoms, setting the scene for future clinical trials in this area. Randomised studies are needed to further test whether certain dietary changes are effective for improving specific symptoms of SLE.

## Introduction

Systemic lupus erythematosus (SLE) is a systemic and chronic inflammatory autoimmune rheumatic disease with common symptoms of fatigue, joint and muscle pains and skin lesions, as well as more severe manifestations affecting kidneys, central nervous system, heart and lungs. Symptoms between patients can be heterogeneous, intermittent and range in severity. The relationship between nutrition and SLE is not well established. Quality of the diet is important in the management of patients with SLE because they have a higher risk of developing cardiovascular diseases (CVD), one of the leading causes of death in this population.^(1)^ As a result, dietary regimens for patients with SLE are mainly aimed at reducing CVD risk. However, recent evidence suggests that healthier lifestyle habits could also improve inflammatory markers and immune function, with possible benefit on many disease symptoms.^(2, 3)^

Unhealthy dietary patterns may contribute to the development and course of SLE.^(2, 4)^ Studies have reported a high prevalence of overweight and obese patients with SLE, ^(5, 6)^ and overweight status has been associated with the presence of nutrient deficiencies and increased disease activity.^(2, 5, 7)^ A study examining self-reported dietary intake in patients with SLE revealed that patients had diets low in fruits, vegetables and dairy products, but high in oils and fats.^(6)^ Unhealthy diets may lead to a disturbed gut microbiome and breached intestinal barriers, allowing undigested food proteins into the circulatory system and activating the immune response.^(8)^ Antibodies formed against food-specific antigens could then cross-react with human tissues leading to the development of extra-intestinal autoimmune diseases.^(8, 9)^ In a recent study, antibodies against food antigens commonly found in industrial processed foods including wheat (gluten), milk, peanuts, soy, egg and corn all demonstrated cross-reactivity with human tissue proteins.^(8)^ Anti-gluten antibodies may have sequence homologies and cross-react with brain antigens, pointing to a possible pathway between Western diets and neurodegenerative, neuropsychiatric and neuroinflammatory diseases.^(10)^ Gluten also appears to contribute to the tight junction dysfunction and intestinal permeability associated with initiation of the autoimmune cascade.^(11)^ Avoiding gluten-containing foods could improve symptoms of non-Celiac autoimmune diseases by reducing leaky gut, inflammation and the initiation of autoreactivity. Conversely, daily consumption of fresh vegetables and fruit could have a positive impact on SLE disease symptoms.^(4, 12)^ However, to date, there is insufficient information on diet as a therapeutic strategy to control SLE.

The lack of evidence-based nutritional guidance for patients with SLE highlights the need to explore the impact of different eating patterns on symptoms and other disease markers in this patient population. In a recent UK-based survey, SLE patients expressed interest in using diet to treat fatigue and other symptoms.^(13)^ SLE patients undertaking certain kinds of restrictive eating regimens also reported improved disease manifestations. In the present study, we further investigate the relationship between eating formats and self-reported disease symptoms and general health aspects in SLE by surveying patients on their experiences with SLE and diet. Engaging patients in research in this way will complement clinical and basic studies to maximise research translation for patient benefit.

## Methods

### Study Design

An anonymous online survey was designed to obtain information on patient experiences regarding SLE symptoms and diet. The survey consisted of a lay research summary outlining the study objectives of the survey (Supplementary Methods), followed by 26 questions focusing on patients’ experiences around eating formats and SLE symptoms (Table 1). These were reviewed by nutritionists, consultant rheumatologists and basic science researchers within the Division of Medicine, UCL. The approximate time needed for completing the survey was 5–10 min. There were multiple-choice questions (allowing one answer), checkboxes (allowing multiple answers), rating scales and free text boxes. Research Authority decision tool (https://www.hra.nhs.uk/approvals-amendments/) confirmed that ethical approval was not needed for this survey.

**Table 1.** Online survey questionnaire.

Question	
1	Do you have systemic lupus erythematosus?
2	Do you have another autoimmune disease? If yes, please list which one(s) in ‘other’
3	Which gender are you?
4	How old are you?
5	Where do you live?
6	When you are having a lupus flare, you often feel the desire to
7	Please rate the following statements
	I Take into account my lupus with what I eat
	I Deliberately eat or avoid certain foods in order to control my lupus symptoms
	I Do not eat some foods because they make my lupus symptoms worse
8	How would you rate your level of restraint in eating?
9	What would be your reason for changing your eating habits if you decided to do so?
10	What reasons would prevent you from changing your eating habits in the future?
11	Please rate how severe your lupus symptoms were at their worst ever
12	Would you consider changing your eating habits if you were told it could help with your lupus symptoms?
13	Have you already tried changing your eating habits since you were diagnosed with lupus?
14	Why did you decide to change your eating habits?
	Please check all that apply and explain in ‘other’
15	How did you change your eating habits? Please explain
16	Did your new eating patterns match any of the following?
	Please check all that apply
17	How long did you undertake this eating pattern?
18	Did you benefit from changing your eating patterns?
	If yes, please explain how in ‘other’
19	Please rate how severe your lupus symptoms were before and after you changed your eating patterns
20	Did changing your eating patterns help with any of the following?
	Fatigue/Weight loss/High cholesterol/Nausea/Rashes skin lesions/Hair loss/Mouth ulcers/Joint or muscle pain/Fever or chills/Headaches/Shortness of breath/Sleep/Mood/Decreasing medication/Other (please specify)
21	If taking medication, has your medication changed since you started your new eating patterns?
	If yes, please explain how
22	If taking medication, did your medication affect your eating patterns?
	If yes, please explain how
23	Typically, do you eat… (Please check all that apply or explain in ‘other’)
24	How many hours in between your last meal 1 day and first meal the next day? (Ex. Between Mon dinner and Tue breakfast.)
25	If you’ve taken supplements since your lupus diagnosis, which ones?
	Please check all that apply or list in ‘other’
26	What is your current height and weight?

An anonymous online survey consisting of 26 questions asked SLE patients about their eating patterns and disease symptoms, including beliefs and experiences.

### Response Capture

The survey was hosted online on Survey monkey (www.surveymonkey.com) for around 4 weeks (13th February to 16th March 2020). Lupus UK, Centre for Adolescent Rheumatology versus Arthritis at University College London (UCL) and other SLE awareness groups helped to promote the survey on Twitter, Facebook and Instagram. Multiple submissions from the same participant were prevented. Although this was an exploratory study acquiring as many responses as possible, a threshold for number of responses captured was set using a proportional power calculation of SLE patients that had (64.18%) and had not (35.82%) been on a diet from our previous study.^(13)^

### Response Analysis

An initial question asking patients to report their diagnosis of SLE was put in place to stratify responses. Only patients with SLE were included in the analysis. Incomplete surveys were included. A single researcher was responsible for collecting and analysing the questionnaires. Data was extracted from SurveyMonkey to Microsoft Excel 2010.

### Statistical Analysis

Statistical analyses were conducted using Social Science Statistics calculator (https://www.socscistatistics.com/) and GraphPad Prism 9. Chi square tests were conducted to compare proportions of respondents. Paired Wilcoxon Signed-Ranks Tests and a Paired-Samples T-test were conducted to compare self-reported disease severity scores before and after dietary change.

## Results

### An online survey promoted through social media is a fast and effective way to gather patient dietary information in SLE

A 26-question survey was made available online for 4 weeks to understand patients’ attitudes and experiences regarding their eating patterns and SLE disease symptoms (Table 1). Among the 458 responses received, 420 (92%) reported their diagnosis of SLE and were used for data analysis. Half of these respondents with SLE reported having another autoimmune disorder; these patients were included in the analysis. Most SLE respondents were female (95%), living in the United Kingdom (90%) and above the age of 24 (92%). There was an even spread of respondents across the 25–65+ years age groups, with patients over 45 providing the most responses (61%). The average body mass index (BMI) was 27.3 kg/m^2^. Most patients were either overweight (28%) or obese (29%), while 39% of patients had a ‘healthy’ BMI (Table 2).

**Table 2.** Demographic information of survey responders with SLE.

Demographic information	Number (%)
Gender	372
Male	15 (4%)
Female	355 (95%)
Non-binary/Other	1 (0%)
Prefer not to say	1 (0%)
Age (yr)	372
Under 18	3 (1%)
18–24	24 (6%)
25–34	54 (15%)
35–44	60 (16%)
45–54	106 (28%)
55–64	75 (20%)
65+	50 (13%)
Residence	376
United Kingdom	336 (89%)
Europe	12 (3%)
North America	14 (4%)
Central/South America	0 (0%)
Asia	3 (1%)
Africa	3 (1%)
Australia/New Zealand	4 (1%)
Other (please specify)	4 (1%)
Other autoimmune disease	376
Yes	186 (49%)
No	190 (51%)
Body Mass index (BMI) (kg/m^2^)	345
>30	100 (29%)
24.9–29.9	97 (28%)
18.5–24.9	135 (39%)
<18.5	12 (3%)

The table displays the percentage and number of survey responders by gender, age, place of residence, presence or absence of another autoimmune disease and body mass index. All parameters are self-reports. The standard weight status categories associated with BMI ranges for adults are >30 kg/m^2^= Obese, 24.9–29.9 kg/m^2^= Overweight, 18.5–24.9 kg/m^2^=Normal or Healthy Weight, <18.5 kg/m^2^= Underweight.

### SLE symptoms influence food choices and patients are willing to change their eating patterns to help with symptoms

**Figure 1.**
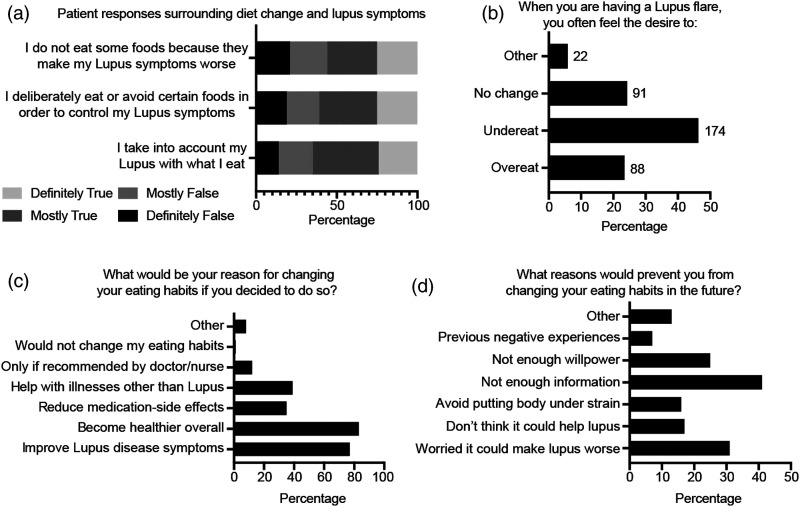
Patients’ beliefs on the link between diet and their SLE symptoms. **(A–D)** Data is displayed as the percentage of total patients that responded to the question.

Most patients reported that their SLE symptoms affected their food choices (Figure 1(a)). This included considering their symptoms with regard of what they eat (65%), deliberately eating/avoiding certain foods to control symptoms (61%) and not eating some foods because they make symptoms worse (57%). In addition, most patients expressed that their SLE symptoms impacted their appetite (Figure 1(b)), with 46.4% more likely to under-eat than over-eat (23.5%, *p*<0.001) or not change the amount they eat (24.3%, *p* =0.014) when having a disease flare. Thus, SLE symptoms appear to decrease patients’ appetite and influence their dietary choices. With this respect, the main reasons for dietary changes were to become healthier overall (83%) or to improve lupus symptoms (77%) (Figure 1(c)). In contrast, common reasons reported for not changing eating habits were due to not having enough information (41%), worrying it could make their lupus symptoms worse (31%) or lacking willpower (25%) (Figure 1(d)). All but one respondent (99.7%) indicated that they would consider changing their eating habits if told it could help with their lupus symptoms. Thus, SLE patients are willing to alter their eating formats, but many feel that they lack the information to do so.

### A majority of SLE patients have attempted dietary modification, which commonly resulted in patients being less symptomatic

**Figure 2.**
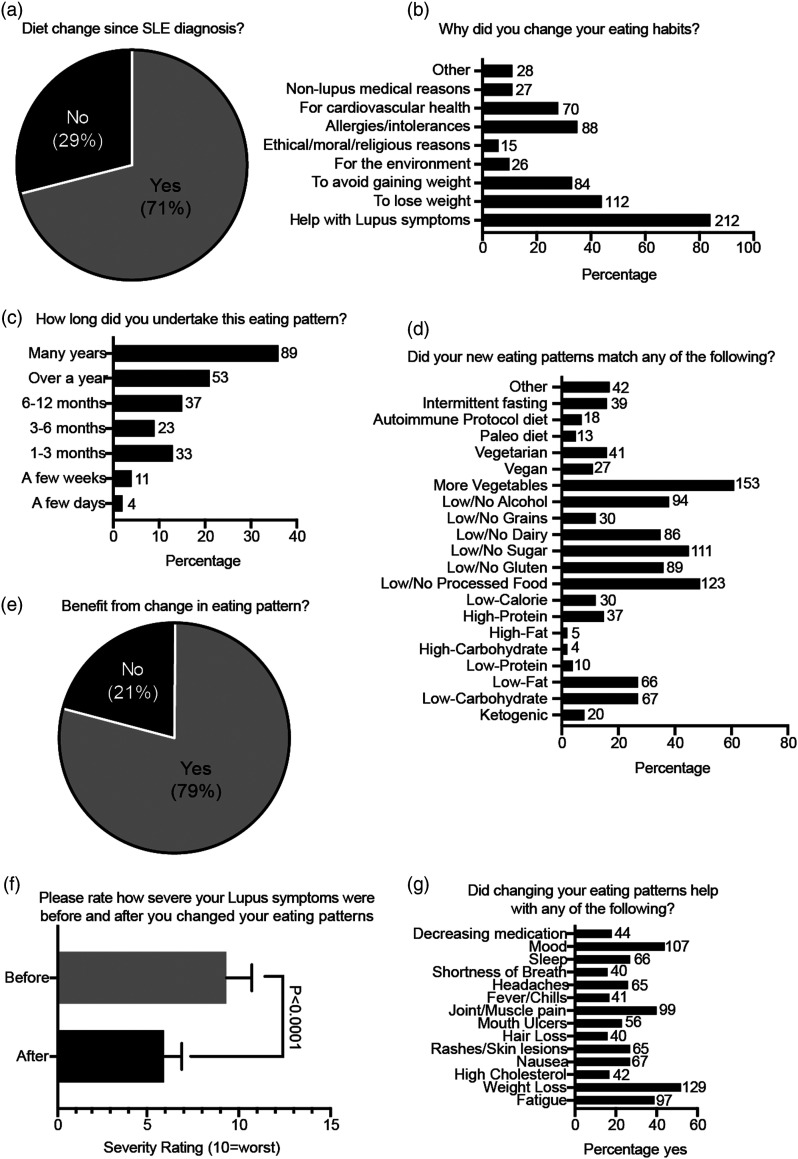
Patients’ experiences with dietary change and their SLE symptoms. **(A–E)** Data presented as the percentage of total patients that responded. **(F)** Paired T-Test, Mean and SEM. **(G)** Data presented as the percentage of total patients responding ‘yes’. **(B–D, G)** Number of respondents displayed to the right.

Many respondents (71%) said they had already tried changing their diet since their SLE diagnosis (Figure 2(a)). The most common reasons for diet change reported were to help with lupus symptoms (84%), to lose weight (44%), for allergies/intolerances (35%), to avoid gaining weight (33%) and for cardiovascular health (28%) (Figure 2(b)). Over half of respondents (57%) undertook their new eating patterns for over 1 year (Figure 2(c)). The most common changes in eating formats reported were increased consumption of vegetables (62%) and lower/no consumption of processed food (50%), sugar (45%), alcohol (38%), gluten (36%) and dairy (35%) (Figure 2(d), Supplemental Page S2). Many patients also undertook low-carbohydrate (27%), low-fat (27%), vegetarian (16%) and vegan (11%) eating formats.

Most respondents (79%) undertaking a dietary change indicated that they benefited from their new eating formats (Figure 2(e)). Importantly, symptom severity ratings after dietary change were significantly lower than before (21.3% mean average decrease, *p*<0.0001, Figure 2(f)). When asked about life/symptom improvements after changing their eating habits, patients reported benefit with weight loss (52%), improved joint/muscle pain (49%), mood (43%), fatigue (39%) sleep (27%), nausea (27%) and rashes/skin lesions (27%) (Figure 2(g)).

These findings indicate that patients have tried various eating formats to help with their SLE symptoms with various improvements following dietary modification; most patients included more vegetables in their diet and maintained their dietary change long-term**.**

### Undertaking diet change for longer periods and having greater restraint towards food were associated with greater improvements in the severity of SLE symptoms

**Figure 3.**
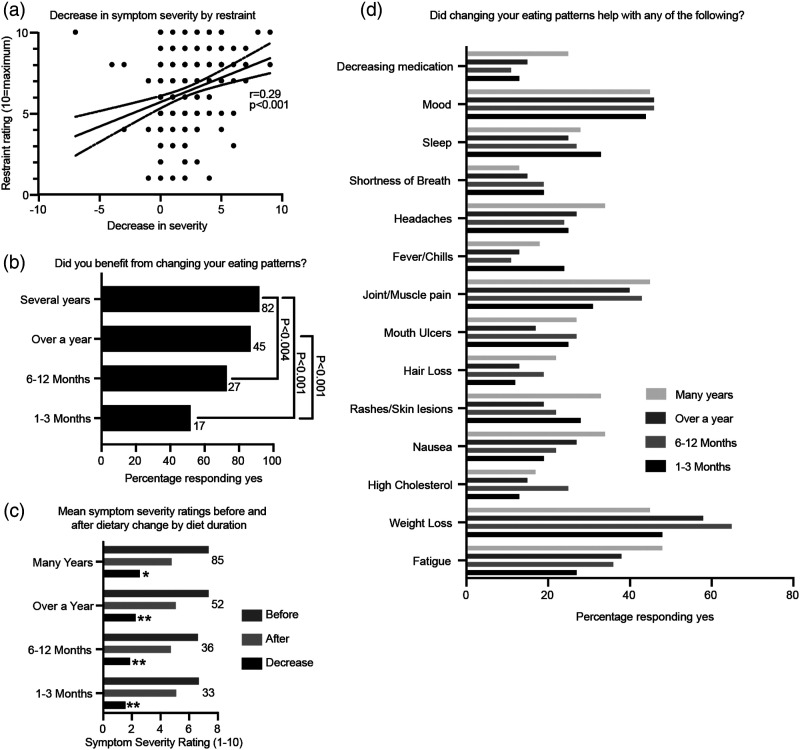
Patients’ symptom benefits by dietary restraint and length. **(A)** Scatter plot demonstrating patients’ decrease in symptom severity by their ratings for restraint in eating. Positive *x*-axis values indicate improvement in symptoms after dietary change. Pearson correlation. **(B)** Percentage of patients that benefited from dietary change for different diet durations. Number of patients that responded to each option is displayed to the right. Chi-square test. **(C)** Mean symptom severity ratings before and after dietary change and the decrease in symptom severity by duration of dietary change. Mean. Paired Samples Wilcoxon Signed-Rank Test; *p<0.001; **p<0.0001. **(D)** Percentage of patients undertaking eating patterns for different durations that responded ‘yes’ when asked whether their new eating patterns helped with specific symptoms.

Dietary restraint and duration were also considered in the context of disease severity. Respondents indicated that they practiced moderate restraint towards food, with a mean average rating of 5.63/10. There was a significant positive correlation between restraint rating and decrease in symptom severity after dietary change (r=0.29, *p*<0.001, Figure 3(a)). In support, most patients that benefited from a dietary change had undertaken their diet for several years (92% benefitted) or over a year (87% benefited, Figure 3(b)). Interestingly, 72% of patients with dietary change duration of 6–12 months and 52% of patients with dietary change duration of 1–3 months also reported a benefit; however, patients undertaking longer dietary changes were statistically significantly more likely to benefit. Importantly, longer diet durations were also associated with greater improvements in self-reported SLE symptom severity (Figure 3(c)), with the greatest decrease after dietary changes lasting several years (25.8% decrease, *p*<0.001), followed by over a year (23% decrease, *p*<0.0001), 6–12 months (18.9% decrease, *p*<0.0001) and 1–3 months (15.8% decrease, *p*<0.0001). Specifically, as the duration of dietary change increased, a greater percentage of respondents reported help with fatigue (Figure 3(d)), with 48% of patients reporting improvement with dietary change for several years compared to 27% of those with dietary change for 1–3 months. Patients following a dietary change for several years also reported greater help with joint/muscle pain, hair loss, headaches, nausea and decreasing medication than those following one for 1–3 months. However, more respondents following a dietary change for 6–12 months reported weight loss (65%) than those following a dietary change that lasted for several years (45%). The percentage of patients reporting help with mood was relatively equal across dietary lengths (44–46%). Finally, a greater percentage of patients undertaking dietary change for 1–3 months reported help with sleep, shortness of breath and fevers/chills than those with longer diet durations.

Therefore, dietary restraint is associated with disease specific benefits and, while short diet durations may help with some SLE symptoms, patients undergoing dietary change for longer durations are more likely to see improvements in symptoms such as fatigue and pain.

### Dietary changes involving more vegetables, but fewer processed foods and animal products may be of greatest benefit for reducing the severity of SLE symptoms.

**Figure 4.**
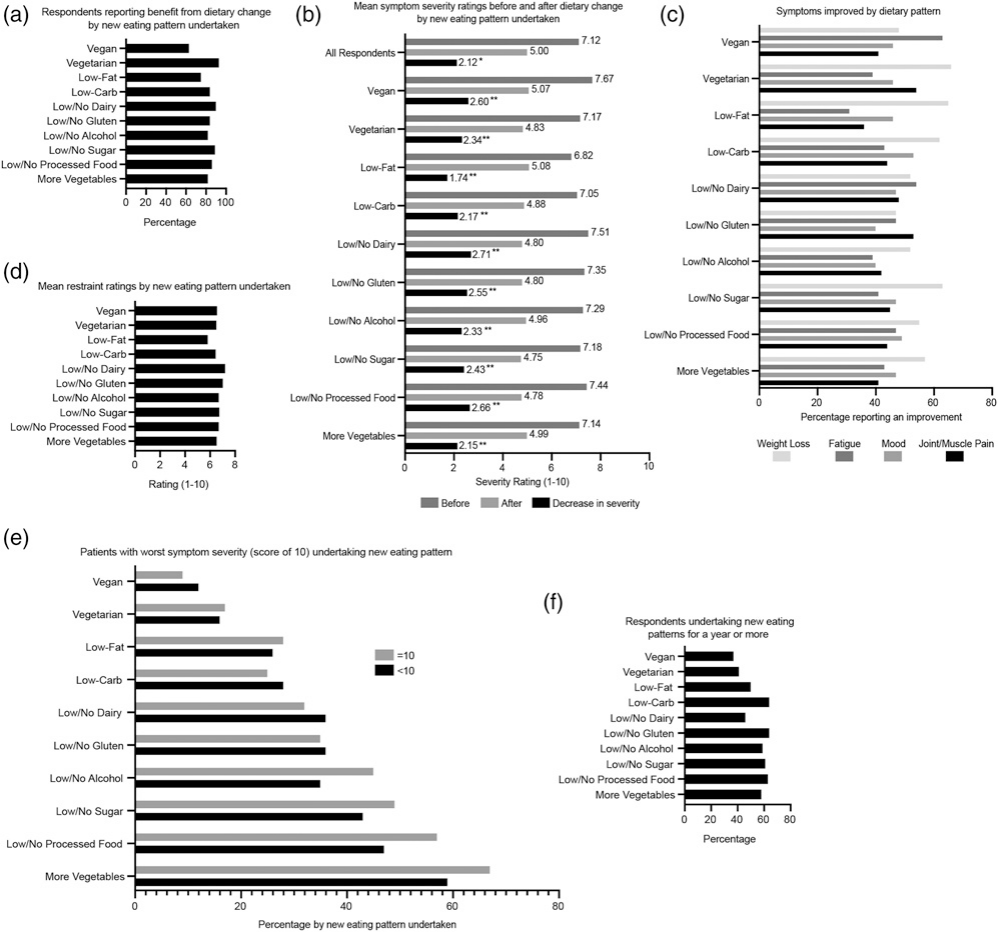
Patients’ symptom benefits from specific new eating patterns undertaken. **(A)** Percentage of patients following a certain eating pattern that responded ‘yes’ when asked if they benefited from their dietary change. **(B)** Patients’ mean symptom severity ratings before and after dietary change by type of new eating pattern undertaken. The difference between these values is displayed as ‘decrease in severity’. Mean. Paired t-test (all-patients) and Paired Samples Wilcoxon Signed-Rank Test; * *p*<0.0001, ** *p*<0.00,001. **(C)** Percentage of patients responding ‘yes’ to whether their new eating patterns helped with fatigue, weight loss, mood or joint/muscle pain by type of new eating pattern undertaken. **(D)** Mean ratings for restraint in eating by new eating pattern undertaken. 1–10 rating scale, 1 = no restraint, 10 = total restraint. **(E)** Percentage of patients with the worst symptom severity ratings (score of 10) or score<10 undertaking each new eating pattern. **(F)** Percentage of patients undertaking dietary change for more than a year by the type of eating pattern undertaken.

The majority (>80%) of respondents that undertook new eating formats with increased vegetable intake and/or decreased intake of processed food, sugar, gluten, dairy and carbohydrates reported benefiting from their dietary change (Figure 4(a)). Vegetarian eating patterns provided the most self-reported benefits (93% benefited), whereas vegan diets provided the lowest, but still substantial, proportion of positive responses (63% benefited).

All eating patterns examined had significantly lower symptom severity ratings after dietary change than before (*p*<0.0001) (Figure 4(b), Supplemental Table S1). The greatest decreases in symptom severity was provided by low/no dairy (27.1% decrease), low/no processed foods (26.6% decrease) and vegan (26% decrease) eating patterns (*p*<0.0001). However, respondents undertaking these eating patterns also had the highest symptom severity ratings before their dietary change. Patients undertaking low/no gluten, low/no sugar diets and low/no alcohol eating patterns displayed larger decreases in symptom severity than the average reported by all respondents. These findings suggest that although diverse dietary changes are associated with SLE patients feeling less symptomatic, eating formats involving more vegetables and fewer processed foods, carbohydrates and animal products are more likely to be of benefit for patients.

Regarding specific symptoms, weight loss, fatigue, joint/muscle pain and mood were the most cited symptoms that improved from dietary changes. Help with these symptoms was more likely associated with certain dietary changes (Figure 4(c)): For weight loss, the highest percentage of patients indicating improvement undertook vegetarian (66%), low-fat (65%), low/no sugar (63%) and low-carb (62%) eating patterns; For fatigue, improvement was highest with vegan (63%) and low/no dairy (54%) eating patterns; For joint/muscle pain, improvement was highest with vegetarian (54%) and low/no gluten (53%) eating patterns; For most eating patterns examined, 40–50% of respondents indicated that their dietary change helped with mood; however, a low-carb diet had a slightly higher percentage (53%). This suggests that dietary changes involving less animal products and carbohydrates are more effective for patients with SLE who wish to lose weight, increase energy, decrease joint/muscle pain or improve mood.

Ratings for restraint in eating were highest on average for respondents with low/no dairy (7.22/10) and low/no gluten (7.03/10) eating formats (Figure 4(d)). Importantly, restraint ratings were also relatively higher for patients with more vegetables, low/no processed food, low/no sugar, low/no alcohol, vegetarian and vegan eating formats. In addition, a larger percentage of respondents with the highest symptom severity (rating of 10) undertook diets involving more vegetables, low/no processed food, low/no sugar and low/no alcohol compared to respondents with lower severity ratings (Figure 4(e)). Finally, the majority of respondents that followed low/no gluten (64%), low-carb (64%), low/no processed food (63%), low/no sugar (61%), low/no alcohol (59%) and more vegetables (58%) eating formats undertook their dietary change for over 1 year **(**Figure 4(f)). However, fewer than half of patients with vegan (37%), vegetarian (41%), low/no dairy (46%) and low-fat (50%) eating formats reported dietary change duration of over 1 year**.** These findings suggest that diets limited in animal products may be difficult for patients to adhere to and increasing vegetables, while restricting carbohydrates and processed foods, could be a more sustainable dietary approach long-term.

Together, this data suggests that increasing the quantity of plant-based foods in the diet, while decreasing processed foods and sugar, is especially beneficial for decreasing SLE symptoms long-term, especially in patients with self-perceived active lupus.

## Discussion

This survey provides insight into the impact of dietary choices on self-reported disease symptoms and general aspects of health in patients with SLE. There were three key observations: 1) SLE influences most patients’ appetite and dietary choices; 2) dietary modification is likely to result in patients feeling less symptomatic; 2) dietary restraint and changes undertaken over the long-term could increase the clinical benefit to SLE patients, and this data sheds light on patients’ ability to adhere to certain eating patterns; 3) our findings highlight the benefits of a whole-foods, plant-based diet (WFPB) – one that incorporates large amounts of vegetables while limiting processed foods, sugar, red meat and dairy products – to improve symptoms in SLE patients.

Many patients with chronic conditions have adjusted their eating formats for their disease, and evidence suggests that individuals are more likely to eat well if they believe it will impact their disease symptoms.^(14)^ Almost all our survey respondents were willing to change their eating formats if told that this would help with SLE symptoms. However, our findings also indicated that lack of information is a barrier to patients trying diet as an adjuvant therapeutic option, as previously reported.^(13)^ Prior research has highlighted benefits of personalised diet with respect to reducing symptoms in patients with SLE.^(4)^ Most of our respondents had already tried changing their eating formats to help control their disease. Of those that had, most reported that their dietary change had improved their SLE symptoms and the average severity of symptoms after dietary change was significantly lower compared to before*.*

There is strong evidence that WFPB diets are associated with decreased risk of major chronic diseases.^(15, 16)^ Thus, recent guidelines for healthy and sustainable eating encourage inclusion of more plant-based over animal foods for overall health benefit.^(15)^ Although some WFPB diets are vegan or vegetarian, the Mediterranean Diet (MD) is a popular variation associated with positive health outcomes that admits animal-derived foods in small quantities.^(16, 17)^ In our study, respondents who reported adhering to WFPB eating formats were more likely to experience benefits from dietary change, including significant decreases in SLE symptom severity, especially for SLE patients with initially severe symptoms. WFPB diets have been associated with improvements in chronic low-grade inflammation, including lower serum C-reactive protein, fibrinogen and total leucocyte concentrations,^(18, 19)^ perhaps due to the anti-inflammatory actions of high dietary fibre. In a recent study, greater consumption of MD foods such as vegetables, fruits, fish and olive oil and abstinence animal products, sugar and pastries was associated with lower SLE activity, damage and CVD risk.^(20)^

Conversely, large intakes of compounds found in processed foods such as sugar and gluten is associated with gut dysbiosis, systemic inflammation and exacerbation of SLE symptoms.^(4, 7, 21, 22)^ Petric et al. found that SLE patients in clinical remission who often ate meat, fast food or fried foods had lower levels of C3 than patients who had high intake of vegetables, fruit and fish.^(12)^ Interestingly, substituting a serving of red meat with alternative protein sources (including poultry, fish, legumes or nuts) has been associated with lower CRP levels.^(23)^ In a recent systematic review, Alwarith et al.^(24)^ observed that diets abundant in vegetables, fruits and fibre reduced joint pain in RA, while diets rich in meat and dairy exacerbated joint inflammation. Like gluten, cow’s milk has high antigenicity and could compound the risk for developing autoimmune disorders.^(25)^ A diet free of gluten, dairy and meat may improve auto-immune symptoms via a reduction in immune-reactivity to these food antigens.^(26)^

Other mechanisms by which WFPB diets could improve SLE symptoms include promoting weight loss, decreasing intake of pro-inflammatory compounds found in processed foods and meats and increasing intake of anti-inflammatory plant metabolites (Supplemental Table S2). Fibre and omega-3 fatty acids have repeatedly been linked to improved outcomes in SLE patients with respect to reducing oxidative stress, inflammation and disease severity.^(2, 4, 27)^ Vitamins C, E and fish oil have also shown efficacy for diminishing oxidative stress and pro-inflammatory cytokines in SLE patients.^(28, 29)^ Thus, the administration of ‘nutraceuticals’ in the form of food derivatives may also have promising outcomes in autoimmune disorders.^(30)^ However, more research is needed to explore underlying mechanisms of dietary modulation and supplements on disease activity in SLE.

Dietary changes led to weight loss for many of our survey respondents. Excess weight is a risk factor of inflammation and has been linked to worse symptoms and quality of life in patients with SLE.^(5, 7, 31)^ A reduction in white adipose tissue can decrease circulating inflammatory mediators and improve SLE disease related outcomes.^(32)^ Vegetarian eating formats and those restricting fat, sugar and carbohydrates provided the highest percentage of patients reporting weight loss. In support, vegetarian diets have shown significant benefits for weight reduction compared to non-vegetarian diets in a multitude of interventional trials.^(33, 34)^ Dietary manipulation and associated weight loss may also help the management of fatigue in patients with SLE, where many of our respondents reported improvements following dietary change. Fatigue affects up to 80% of patients with SLE and is one of the most common and disabling symptoms.^(35)^ In many dietary intervention trials, levels of fatigue have been inversely related to weight loss, irrespective of the type of dietary intervention.^(4, 36)^ Vegan and dairy-restricted eating formats provided the most benefit for fatigue in our study and prior case-studies have linked vegan diets to increased energy in SLE patients.^(37)^ Furthermore, in patients with other chronic conditions such as multiple sclerosis, fibromyalgia and rheumatoid arthritis (RA), vegan or vegetarian diets resulted in improved fatigue and weight loss indices when compared to omnivorous diets.^(38)^ However, the effects of these diets are still uncertain due to the small sample sizes and moderate to high risk of bias of these studies. Standardised reporting measures for fatigue and agreement on a threshold indicating benefit are needed for meta-analyses comparing the impact of dietary interventions on fatigue.^(39, 40)^

Levels of restraint in eating have been related to an improved ability of individuals to control their weight without negative effects such as developing eating pathology.^(41)^ However, prior studies have found no significant differences in restraint among different therapeutic diets.^(42)^ WFPB eating patterns were associated with higher levels of restraint than the average reported by all respondents and higher levels of restraint in eating were positively correlated with greater improvements in SLE symptom severity. Interestingly, even short-term dietary changes helped with sleep, shortness of breath and fevers and chills. Although long-term dietary changes were most associated with decreases in fatigue, joint/muscle pain and medication requirements, both long and short-term dietary changes also led to mood improvements. This suggests that encouraging dietary changes of any duration is a promising approach for reducing SLE patients’ symptoms, but that sustainable long-term changes are most likely to be beneficial. With this respect, it is important to note that not only the food type is important but also the pattern of intake of the dietary change, as suggested in other inflammatory diseases during fasting.^(43, 44)^

There were several limitations in our study. Firstly, since this study was anonymous and online, we were not able to follow-up patients or examine medical records to confirm disease severity or treatment in the context of patients’ diet; we therefore relied on patient reported symptoms and disease experiences. Our survey was targeted at specific SLE patient support sites, validated by the proportion of female survey respondents (95%) and highest response age between 45–64 years, representative of SLE epidemiology.^(45)^ Analysis of self-reported symptoms is not uncommon in SLE research,^(46, 47)^ however, in the future it will be necessary to validate these findings with objective data in a clinical setting.^(40, 48)^ Publishing both patient reported and clinical outcome measures is crucial to maximising the translational potential of research for patient benefit. Secondly, this survey was restricted to English speaking patients with internet access and patients may have chosen to take the survey based on a prior interest in the topic. Since most respondents were living in the UK, these results may differ for SLE patients residing in countries where diets, food availability and quality are different. In future, we would consider translating the survey to reach a wider patient population. Thirdly, in some cases, certain questions were skipped, as reflected in our completion rate; survey-taking fatigue may have accounted for this, suggesting the survey may have been too long for some patients. Finally, lifestyle changes that accompany dietary modification could have confounded the relationship between diet and symptomatic benefit, including physical activity, abstinence from smoking and alcohol and stress-management techniques.^(49)^ Finally, highly symptomatic patients may be more likely to engage in lifestyle changes, as suggested by their greater likelihood to undertake WFPB eating formats in our study. More extreme dietary or non-dietary alterations could explain why patients with greater initial symptom severity had greater improvements in their symptoms.

## Conclusion

This study suggests that diet intervention could play a role in decreasing the severity of symptoms of patients with SLE and improving general health, with a WFPB diet appearing to have the most beneficial impact in our patient group. Disease activity in SLE could be influenced by eating formats involving large amounts of processed foods, sugar, meat and dairy products. Limiting these pro-inflammatory food sources while augmenting anti-inflammatory, plant-based ones may improve SLE symptoms in patients. The willingness and interest of SLE patients in changing their eating patterns highlight the potential for interventional clinical trials; this would help establish the effects of dietary interventions to provide the evidence-based information required to inform patient choices and educate healthcare providers to offer adequate guidance to improve the health and quality of life for patients with SLE.

## Supplemental Material

sj-pdf-1-lup-10.1177_09612033211063795 – Supplemental Material for Plant-based dietary changes may improve symptoms in patients with systemic lupus erythematosus Click here for additional data file. Supplemental Material, sj-pdf-1-lup-10.1177_09612033211063795 for Plant-based dietary changes may improve symptoms in patients with systemic lupus erythematosus by Aziyadé Knippenberg, George A Robinson, Chris Wincup, Coziana Ciurtin, Elizabeth C Jury, and Anastasia Z Kalea in Lupus
