# Supplementary material for: Plant-based dietary changes may improve symptoms in patients with systemic lupus erythematosus
Source: Lupus. 2022 Jan 3;31(1):65–76. doi: 10.1177/09612033211063795 (PMC8793314; doi:10.1177/09612033211063795)
Supplement: sj-pdf-1-lup-10.1177_09612033211063795 – Supplemental Material for Plant-based dietary changes may improve symptoms in patients with systemic lupus erythematosus [file sj-pdf-1-lup-10.1177_09612033211063795.pdf]

Supplementary Methods

Lay Research Summary

How you can help influence a new research proposal.

We are a group of researchers at University College London (UK) studying patients with Lupus. We want to increase our understanding of what causes the disease so that we can improve and develop new treatments for patients. We have a new and exciting idea that we believe could help to reduce disease severity. Before we start this project, we would like to find out from you whether you have any experiences that could help us improve our research.

We are interested in how diet can influence the immune system (the body's natural defense system). We have found that certain foods are linked with harmful inflammation in the body and disease flares. We would like to find out whether altering the diet could have a beneficial effect on Lupus by decreasing inflammation. Therefore, greater knowledge of how diet has affected patient experience with Lupus would help us understand the potential of this new idea. We hope this approach could reduce the dependence of patients on drugs.

Before we begin this research, we would like to ask you some short questions to help us understand your experience with diet and Lupus. Your input is of huge value to our research. Please click "Next" below to answer these questions.

All of us at UCL Centre for Rheumatology Research would like to extend a grateful thank you for your time and support in answering these questions; it is only together we can really make progress in understanding this disease. By completing this questionnaire, you are consenting to us sharing your responses. All of your answers will be completely anonymous.

Supplementary Data

Free-text responses to: *How did you change your eating habits? Please explain.*

1. *Changed to a low carb diet*
2. *Restricted red meat and cut out spicy and acidic foods. Also omitted fizzy drinks.*
3. *Saw a nutritionist who prescribed an exclusion diet and used paleo diet - gluten free dairy free plus plenty of good protein omega 3 leafy green vegetables. I kept a food diary. Avoided bought gluten free foods as full of additives and sugar. I avoided sugar and this significantly reduced inflammation*
4. *Avoid eating mushrooms. Take meal replacements protein shake for dinner to reduce food intake*
5. *By avoiding certains kinds of food and adding more of others.*
6. *fasting*
7. *Anti-inflammatory diet*
8. *Cut out carbs completely and all meat except fish.*
9. *Generally eat a mediterranean diet. Lowered levels of processed foo*
10. *Went on the autoimmune protocol*
11. *cut out alcohol& caffiene reduced sugar content*
12. *Reduced salt, potassisium intake,*
13. *Reduced sugar and carbohydrate intake.*
14. *Gluten free more plant based less processed foods*
15. *Eating more healthy, less fatty foods*
16. *Tried to eat more healthily and avoid immuno boosting foods*
17. *Went Vegan (for over 3 years)*
18. *Paleo diet, vegan diet, AIP, keto diet with IF, no dairy, no coffee, more turmeric w/black pepper, mire ginger, green tea, etc*
19. *I don't red meat. I've cut down a lot on sugar and caffeine. Tried not to eat gluten.*
20. *I'm just starting and have reduced all sugar, dairy and wheat ! However there is a difficulty with my teeth so I can chew properly caused by the lupus*
21. *Less junk, more nutrients. Healthy food makes me less fatigued and experience less pain*
22. *I'd say I ate healthily anyway however as a family we made home cooked meals and avoided high amounts of junk food. I had a big appetite when I first started taking medication for my Lupus. My mum would say if I'm going to eat to feed my increased appetite then I'd be eating healthy things. Soups and nourishing meals.*
23. *Reducing intake if processed foods, less sugar, less salt, using organic as much as possible, eating less but better quality meat e.g grass fed organic, lots of veg, fish, pulses. Make my own bread so control salt/sugar and no added preservatives. Drink bone broths and take both Vit D and probiotic daily. Although not completely restricted so will eat and go out for occasional drinks.*
24. *I don't eat meat, gluten, soja, milkproducts and limit in fats. I try to eat lots of vegetables, fruits, fatty fish and I start my day with oatmeal.*
25. *Reduced junk food intake, increases fresh fruit and veg, eat less meat and more fish and vegan/vegetarian diet*
26. *Gluten free then lectin free diet*
27. *I stopped eating all gluten based foods and all dairy. I also find animal protein difficult. Since the menopause I have found that I can eat some dairy, especially cheese.*
28. *I eat only meat and animal fat. It has reversed most of my symptoms*
29. *I did try severely reducing sat fat and on different occasion cutting out gluten*
30. *Healthy eating and give up alcohol*
31. *I stopped eating dairy and gluten products.*

32. *Less sugar and processed foods, more veges*
33. *Avoiding some dairy products and spicy food, eating fermented yogurts and more fresh food*
34. *I stopped eating gluten, wheat, eggs & dairy. I have been vegetarian since a teenager but still eat all fish*
35. *Cutting out meat and cutting down on dairy and using dairy alternatives. Also cutting down on sugar*
36. *Cutting out processed foods, bad sugars and carbs.*
37. *I have become completely gluten intolerant which caused extreme pain in my chest and shoulders, I cannot eat blueberries or cranberries as they make me ill to. I don't drink full cream milk or cream as it worsens my asthma*
38. *Less meat. More vegetables. Less processed foods.*
39. *I gave up gluten containing foods. I cut down on lactose, but occasionally have cheese or sour youghurt*
40. *Eating more fruit and veg eating double the amount of food*
41. *Cut out or cut down on carbs*
42. *Reduced gluten, procesed food eat more protein fresh food*
43. *Healthy snacks*
44. *Healthier options in the majority of food input.*
45. *More veg. More water. Less alcohol. Minimise carbs.*
46. *Information from myson who is studying nutrition at uni*
47. *Cut certain items out of diet e.g milk etc*
48. *Less dairy eg use oat milk now. Eat veggie most of the time. Try to limit alcohol on weekdays as much as possible. Eating has become much healthier overall. Less processed food, much more fruit/veg and whole grains, less sugar most of the time. Trying to reduce portion size but that's the hardest thing so concentrate on filling up on veggies. Eat quite a high protein diet at the moment as I've started going to the gym / lifting weights.*
49. *I eat less or no meat, a variety of vegetables,do not eat dairy products*
50. *Cut out gluten and limit dairy.*
51. *Not eating some food that increases my disease, eating more healthy (vegetables, fruits, etc)*

52. I'm now gluten free and dairy free and eat more fruit and veg
53. Reduce sugar, fat and fried, salt, lactose intolerante (milk and yogurts) more fruit and vegetables
54. Avoid all foods I am severly allergic to. I carry epipens for the nut allergy. With advice from gastroenterology and dietician cut gluten completely. Alot of my symptoms continued, muscle weakness, flu like symptoms, joint pain unable to work. Then diagnosed with lupus. Now use information about inflammatory food to help. So desperate to feel better will try anything I am advised once I have investigated information. I am careful because I am determined to keep going.
55. Gluten and dairy free
56. Became diary free and reduced red meat and ate more vegetables
57. I went down to basics, at my worse, each day I introduce/add one food item, for example today I eat plain rice, and monitor any symptoms, then the next day add green beans and monitor. If I find any diverse result I exclude that type of food frolm my diet, until I established my food triggers.
58. Became pescitarian
59. I did cut out dairy products and 90% of glúten, every home made, no industrial product, loads of fibres and fruits, very colourful plate everyday!! Salads, vegetables, use loads of garlics, ginger, turmeric helped with inflammation joints!!!
60. Trying to eat more fruit, vegetables and fish. Reduce my meat in take, also reducing the amount of processed and fast foods I'm eating. Reduce the intake of crisp, sweets and chocolate.
61. Ate less and cut out fatty foods and caffeine and to much sugar as I was worried about drug side effects making me fat especially steroids.
62. Vegan
63. I removed things that cause me issues such as potatoes and eat more pineapple and blueberries
64. Lowered my carb intake and tried to stay away from processed foods and tried to me more active
65. Joined slimming world
66. Slimming world
67. Less salt,sugar and fat avoid meat totally
68. I controlled fats, carbs and proteins. When I exercise I eat brown rice as carbs, protein and greens. I eat more fruit, low fat 0%sugar sugar yoghurt. And I eat lots of green vegetables everyday, 2 with main meal but no carbs. I'be also cut sugar almost completely from diet
69. Gave up meat and processed foods. Just trying to become more healthy and balanced all round
70. Gluten free
71. I try to avoid immune system boosting foods
72. Vegetarian for health and ethical reasons, avoid garlic and alfalfa sprouts because they trigger lupus flares. I limit processed sugar and white flour.
73. Increased calcium intake e.g milk etc
74. Had pancreatitis a few times because of lupus and pbc, so keep to a low fat diet, avoid spicy foods
75. Green smoothies and trying to eat a Plant base diet
76. Avoided buying junk food and processed foods often and cooking more rather than eating out/or ordering takeout
77. Changed to a vegetarian diet with limited process foods
78. Low carb gluten wheat fre no citrus
79. Reduced processed foods
80. I gave up red meat and additional sugar
81. Low salt carbs eat more fruit beg
82. Calorie controlled, lower fat diet.
83. Low salt, cut out red meat, no garlic, avoid nightshade vegetables....upped my fruit and vegetables, try to include salmon once or twice a week, cut out caffine, cut out alcohol, low protein as I have stage 1 kidney disease, I have fruit oats and live yogurt for breakfast....I periodically try to be gluten free
84. Became vegan
85. Vegan
86. cut down on wheat and nightshades (potatoes, tomatoes, peppers, eggplant, paprika...); I also tried the AIP (auto-immune protocol) but I didn't find that it helped that much to be so restrictive
87. Eat less meat, eat more vegetarian meals, eat more fish, eat more vegetables, cut out liquorice and avoid alfafa sports. Reduc e my caffeine levels and increase my calcium levels
88. Reduce foods that affect my lupus or avoid eating totally.
89. Healthier Eating
90. Cut out gluten,hay diet,no deadly nightshade family,no sugar,supplements,probiotics
91. Changed from high cholesterol and red meat, attempted vegan. Now eat balanced healthy diet
92. Ate whole foods, little processes anything.
93. I was already gluten free (since 2005). Diet was Mediterranean but has been whole foods plant based since Lupus diagnosis in July 2018.
94. I cut out garlic, nightshade vegetables, changed potatoes to sweet potatoes. Less coffee too
95. By excluding gluten my chest pain/pericarditis improved significantly. Tomatoes, citrus, onions, garlic, gluten, wine, and too much sugar affect joint pain and chest pain
96. Trying to eat more foods rich in iron as anaemic, made worse by medication.
97. More whole foods, limiting carb intake but NOT Keto and avoiding processed foods
98. Reduce sugar because of steroids, increase calcium (for bone health) try and reduce fatigue with more whole grains
99. I have gone vegan Nov 2019
100. No processed food low intake of sugar
101. Read about it
102. Gluten and wheat free. No red meat, more vegetables and salads more of a Mediterranean diet
103. I tried the auto immune diet, I gave up alcohol and sugar and gave up garlic as made my symptoms worse
104. Followed Slimming World plan
105. Keto
106. Stopped eating dairy
107. Eat less processed foods, less meat, increase vegetables and pulses
108. balance

109. *I went plant based*
110. *I don not consume dairy . I am a bit unsure about certain fruit*
111. *As an adult i was diagnosed with lactose intolerance so I have had to change my diary intake and substitute. I also had to eat a high carb and calorie meals due to chemo and my illnesses causing weight loss and to maintain a healthy weight*
112. *Diet after gaining weight from high dose steroids*
113. *In 1981, World-leading Consultant Rheumatologist, Prof. Graham Hughes, at London's Hammersmith Hospital suggested that I might become Vegetarian...I was living on low-income Incapacity Benefit and only ate a little meat, including, mine, bacon etc...I decided that I was in so much pain with very high inflammatory-markers that it was worth a try. I was diagnosed with Lupus, 1976, became Vegetarian since 1981 and I am happy with this change in diet. My Italian ancestors in central/Southern Italy ate mainly a naturally-Vegetarian diet. These days, this dedication exists also for strong ethical reasons re killing animals to eat meat. I am happy I do not do eat meat or fish and am not taking into my body, 'unidentifiable' material such as as livestock antibiotics etc, which could adversely affect my immune system. I also like the fact, that despite being on a maintenance dose of Prednisolone Stetted, my bodyweight is a constant 9stone.*
114. *I saw a nutritionist, plus did a lot of reading into gut Microbiome and inflammation. I then found that eliminating dairy and gluten from my diet has reduced my overall swelling, plus headaches have gone (apart from if I inadvertently eat cows milk products. I also used to have axillary pain on one side, which only reappears if I eat food with wheat. I avoid the nightshade foods too.*
115. *Carried out York allergy testing as recommended by my local group and gave up dairy, gluten and eggs.*
116. *Less Sugar*
117. *Vegan diet for 18 months*
118. *Don't drink acohol and switched to vegetarian 30 years ago*
119. *Stopped eating as much sugar and saturated fats. Also avoid wheat where possible as this causes my skin to have tiny itchy water blisters and upsets my bowels.*
120. *First organic 'everything' then Low Carb High Fat*
121. *Cut down on eating sugar/butter/high carb also potatoes and peppers some spices eg chilli Spread eating times and not eating late in the evening*
122. *Tried to make healthier choices, almost totally eliminated fast food.*
123. *no dairy or gluten, high fibre,plenty of fruit&veg, no additives and e numbers, little red meat, fish and fowl*
124. *I avoid caffeine, am careful with alcohol*
125. *Gluten free diet*
126. *Stopped eating garlic and alfalfa when read can affect. Diet changed for me by myfather when ill as a child. I kept on with a lesser version of this diet throughout life which I feel is essential to keeping me as healthy as possible. Diet is full of veg and fruit, fresh fish, a little meat (all organic), pulses, seeds, and limit sugars, bad fats, alcohol etc. Lots of water every day (boughtNikken Waterfall purifier).*
127. *Switched to low gluten & FODMAP foods*
128. *Watch how much rather than what it was*
129. *Left out certain foods, followed FODMAP diet*
130. *reduce alliums, garlic, onion, belladonna foods tomatoes etc.*
131. *lots and lots of research but there is no one to guide or talk to. nutritionists charge a fortune*
132. *Slimming world*
133. *Cut down on alcohol. Ate more whole foods*
134. *avoided foods which appeared to make rheumatism symptoms worse eg*
135. *I researched Healing Foods Information for ideas, for e.g. Healing Foods Neil's Yard and the Index on Health Area. I changed my weekly shop for several ingredients that may be of help in reducing inflammation other symptoms I had etc. 2) I had seen a GP and suggested try some low FODMAP to find what might still be causing trouble. 3) I saw a gastroenterologist who suggested that I could try reducing or avoiding skins, nuts and seeds. This three pronged approach has helped me I believe keep mostly out of trouble plus 4) plate size portion*
136. *Eating regularly, less sugar, no fast food*
137. *Gradually moving away from foods that I found hard to digest. It just happened naturally because*
138. *I tried to eat a healthier, more balanced diet. And later on I became vegan.*
139. *Adopted a vegan diet. I am trying to avoid processed food and other foods that cause inflammation in the body. I have significantly increased my daily intake of fruit and veg to between 12-17 portions a day.*
140. *By adding much green into the diet*
141. *Minimised intake of certain foods and stopped alcohol*
142. *Had to stop eating as was being sick all the time then chose to eat less junk food when i felt able to eat*
143. *Joined SlimmingWorld*
144. *Tried vegan*
145. *More fruit and veg, more home cooked food, less salt, no caffeine*
146. *Limiting high calorie foods-eating much healthily*
147. *I avoid phosphate in foods to help control kidney function*
148. *Stopped eating beef, salt, almost no sugar, no fried food. And as less processed as possible. It's worked.*
149. *Eating less cake/biscuits/crisps more fruit, veg and fishg*
150. *Eat healthy fresh juice fresh veg*
151. *Removed the nightshades, reduced lactose and all foods with grains, especially wheat. Reduced sugary foods and drink, reduced lectins, pectins abc foods with high cholesterol. Increased foods with magnesium, B6, B12 & Vit D. Eat more above ground veg, fresh meat and fish. Buy fresh, organic and locally produced goods and cook fresh. Reduced processed foods*
152. *Listened to Goodbye lupus dr and decided to try what she suggested ie going vegan however I haven't fully gone vegan but I've definitely taken processed food out of my diet*
153. *Initially on diagnosis I was carrying about 1 stone more than I should so for longevity of joints etc felt I needed to lose a stone. Over the years I have read various articles regarding auto-immune and lupus and have tried to cut down on sugar, processed food and alcohol. I try to each more "clean"*
154. *Trying to on advice to avoid all meat and carbohydrates, sort of a mediterranean diet*

155. *Cut portion sizes and reduced amount of carbohydrates in my diet.*
156. *Raw vegan*
157. *Cut out sugar as much as possible, and getting my sugar intake from fruit. I also cut dairy out of my diet and I eat less gluten when possible. I also eat less fatty foods*
158. *Not eating dairy, changing some foods to gluten free,wholemeal. Balancing fruit and veg combined with my Sjoegrens,Raynaud's and warfarin intake,increased fish intake,*
159. *I eat fermented foods and natural yoghurt daily. I try to eat more oily fish and multi- coloured vegetables daily. I avoided all gluten for about three months but didn't notice any appreciable difference in my symptoms.*
160. *Reduce dairy/gluten/wheat, diagnosed lactose intolerant, now follow low Fodmap diet*
161. *Tried to cut down on amounts, not eat carbs, up veg/salads and stop low-fat foods*
162. *Changed to vegetarian/vegan diet*
163. *Cut out gluten items down and increased protein intake*
164. *To avoid all food that caused lupus to flare.*
165. *Ate more fish and veg and less bread, pastry, pasta. I have a book on lulus and diet. I talked to others at a group meeting.*
166. *Before I was diagnosed with a Lupus, back in the day, I visited an acupuncturist due to severe rheumatic pain. At that time he was the only person who gave me advice on eating. He advised against eating a high fat/high sugar diet. None of the selection of doctors I had seen, both NHS and private had indicated changing my diet would help. I was also advised against eating grapefruit. I took his advice and together with three weeks treatment my joint pains ceased. I'd love to say I have stuck to his words over the years but I haven't, I went on to develop CKF and am now 18yrs post tx and still I receive little/no advice about diet. There were no dieticians to help at any stage of my treatment, even now there is no renal dieticians for outpatients. The only way I have pick up recent trends about foods to avoid is via forums Lupus UK and the NKF*
167. *More fruit and vegetables less alcohol*
168. *Avoided nightshade vegetables and spicy foods that trigger my lupus*
169. *Eat healthy.*
170. *Excluded some foods like dairy sugar and gluten.*
171. *More oily fish , fruit & veg*
172. *I tried to avoid foods with spices. I joined Slimmimg World*
173. *I tried the AIP diet from Amy Myers. Within 3 days my symptoms had massively reduced. I've been diary and gluten free (mostly) after the 3 month programme. For the last 6 weeks I've been Vegan and gluten free. Sadly some symptoms (rheumatism, psoriasis, fatigue) seem to have returned. I think it may be excessive sugar. I have also had the flu. If I don't improve in a month or two I will try the AIP diet again from the beginning.*
174. *Slightly bigger portions so I could better tolerate medication, increased calcium. Adjusting regularly if experience random & unexpected reaction/allergy.*
175. *Don't drink milk. Eat more nuts fruits and veggies. Smaller meat sizes. Not very many bread/cereal carbs. Increased healthy fats.*
176. *by reading research in books and on line about how food effects your body, so I tried them, and some things worked as far as foods a little bit.*
177.
178. *Cut out meat and ate more f8sh*
179. *No dairy, caffeine and packaged foods*
180. *Introduced more fresh foods and vegetables*
181. *Since being diagnosed with lupus I became more aware of my over all health. I have cut out a lot of unhealthy food and try to eat as much fresh food as possible. I have lost over 4 stone since my lupus diagnosis and seen a general improvement in my health*
182. *gradual changes for reduced intake and less sugar, immediate change with fish oils*
183. *Eating more fruit and veg, less carbs and fatty foods*
184. *Excluded wheat and heavily processed foods*
185. *Reduced meat, reduced gluten, reduced fast food/junk etc. Improved amount of antioxidants in diet*
186. *Tried paleo*
187. *Did what i was told*
188. *By avoiding foods that I have learnt cause an adverse reaction due to SLE eg aubergine and using foods that help eg garlic counters the permanent Candida infections I have*
189. *For my training I used to be a bodybuilder and eating healthy was always easy, so I ate the proper amount of protein, carbs, fats for my bodyweight, I know how to get healthy.*
190. *Gluten free*
191. *Gluten-free, dairy free, low fodmap*
192. *Gluten free porridge pasta and bread*
193. *Remove nightshade, gluten, dairy, seeds, some nuts*
194. *Switched to vegan diet*
195. *Sensible, planned meals. I was able to cook then and enjoyed it*
196. *No gluten, no sugar, no garlic, very little dairy*
197. *Tried to eat less sugar and salt. More fruit and veg*
198. *Went gluten free*
199. *I've limit sugar, red meat, processed and junk foods.*
200. *Don't eat bread, high fat dairy, olive/vegetable oils*
201. *Going vegetarian*
202. *Smaller portions, more fruit and veg, healthier options*
203. *No garlic. Switched dairy milk to soya. Avoid nightshade. Limit carbs with steroids. No processed food at all. Make all my own foods. On FODMAP diet*
204. *I totally avoid red meat & mostly eat a plant based whole food diet.*
205. *By restricting certain foods i.e. stop eating red meat*

206. *I don’t eat later than 6pm if possible and I try not to mix carbohydrates and proteins in the same meal. I found drinking chocolate triggered Lupus symptoms which could last 24 - 48 hours. I avoid pig meat of any kind as it too can trigger Lupus symptoms. All rich foods like cream and some puddings exacerbate my condition.*
207. *Tried to eat healthier and cut down on over eating*
208. *reduced salt, sugar, fat. eat more fruit & veg. cut out processed foods*
209. *I started to reduce high potassium foods and salt intake rapidly since I was diagnosed with lupus. I have also reduced the immunity-giving foods like garlic...*
210. *Slimming world diet*
211. *More raw vegetables and fresh juices*
212. *Flexitarian. Cut out cakes, chocolate. Increased probiotics & prebiotics. Intermittent fasting*
213. *Low Carb High Fat*
214. *Gluten wheat free low carb*
215. *Gluten free. More vegetables, less meat*
216. *Went dairy and gluten free. Reduce processed sugar intake.*
217. *I’ve had to cut out several things, onions, garlic, lentils, chickpeas, etx as all now make me very unwell because of the tablets/ lupsu*
218. *Low salt low sugar because I was on steroids and also low protein low salt to help my kidneys*
219. *Reduce fats and increase fiber and cereals*
220. *Had to increase salt and fluid, can't have caffeine or big meals, no msg due to palpitations and gluten appears to cause major flares of lupus. Follow fodmap diet due to IBS type symptoms*
221. *Stopped eating certain foods. Eating less food*
222. *Joined slimmi g world*
223. *Vegetarian/vegan*
224. *Earing more fish in my diet and less red meat. Eating healthier foods, such as less processed food types. Eat more fruit and veg and nuts*
225. *I try to incorporate healthy high fat foods like salmon and avocados. I tend to not eat many carbohydrates so I have consciously tried to eat more of them to help with weight gain.*
226. *I am now dairy and gluten free*
227. *I reduced sugar and fat*
228. *Eat less , move more*
229. *Tried to cut out gluten for a while but didn’t last long. Tried to avoid chocolate but again not always successful!* □
230. *I cut out as much sugar as I could, bread pasta cakes sweets chocolate etc*
231. *From my .early 30s: long term Low FODMAP exclusion diet & 2 years ago exclusive Enteral Elemental 028 Extra*
232. *Avoid egg as believe feeds EBV*
233. *I became gluten free and avoid all inflammatory foods following the book The Autoimmune Solution by Dr Amy Myers*
234. *Gluten free as coeliac, anti inflammatory/Mediterranean diet as much as possible but allow myself treats so I don’t go mad. Healthy balanced diet. More oily fish.*
235. *I have IBS which flares when I eat red meat and some vegetables like cucumber & aubergine. I now only eat chicken, fish & vegetarian meals*
236. *Started to avoid gluten and dairy where possible, and ate more fruit and veg and other nutritious foods, however courses of prednisolone have caused 3+ stone weight gain*
237. *I stopped having dairy due to becoming lactose intolerant. Possibly related to lupus but not confirmed*
238. *Increased calories, nutritional drink and generally less sugary, processed foods*

Supplemental Table 1. Impact of various types of diets on self-reported symptom severity.

	Mean			Median				
	Before	After	Decrease	Before	After	N	Statistic	P-value
							Wilcoxon Test (Paired)	
More Vegetables	7.14	4.99	2.15	7	5	146	Z= -8.7656	<0.00001
Low/No Processed Food	7.44	4.78	2.66	8	5	119	Z= -8.7427	<0.00001
Low/No Sugar	7.18	4.75	2.42	7.5	5	106	Z= -7.7443	<0.00001
Low/No Alcohol	7.29	4.96	2.33	8	5	90	Z= -7.1842	<0.00001
Low/No Gluten	7.35	4.8	2.55	7.5	5	86	Z= -6.9576	<0.00001
Low/No Dairy	7.51	4.8	2.71	8	5	84	Z= -7.2457	<0.00001
Low-Carb	7.05	4.88	2.17	7	5	65	Z= -5.7556	<0.00001
Low-Fat	6.82	5.08	1.74	7	5	61	Z= -4.9835	<0.00001
Vegetarian	7.17	4.83	2.34	7	5	41	Z= -5.0745	<0.00001
Vegan	7.67	5.07	2.6	8	5	27	Z= -3.9276	<0.00001
							Paired-Samples T-Test	
All respondents	7.12	5	2.13	7	5	243	t (242) =15.7, 95%CI (1.86-2.39), SE=0.135	<0.0001

Supplemental Table 1. Decreases in symptom severity ratings before and after dietary change were statistically significant for all survey respondents overall and for each new eating pattern presented. Results were significant at p<0.05.

Supplemental Table 2. How whole-foods, plant-based (WFPB) eating formats may decrease SLE symptoms.

	Mechanism[1]
**Weight loss**	High intake of fibre and water reduces the caloric density of the overall diet while increasing satiety and energy expenditure (1).<br>Resultant decrease in white adipose tissue (WAT), an active and inflammatory organ that releases adipokines, which are molecules that contribute to inflammation in rheumatic disease (2).
**Restricting processed foods, refined carbohydrates and sugar**	Commonly used industrial food additives such as gluten, glucose, salt and emulsifiers breach the integrity of the intestinal-epithelial barrier, resulting in entry of foreign immunogenic antigens and activation of the autoimmune cascade (3).<br><br>Regular consumption of excess free fructose contributes to intestinal accumulation of advanced glycation end-products that cross the intestinal-epithelial barrier and promote inflammation in tissues (4).<br><br>High intake of processed foods leads to excess calorie intake and weight gain and is associated with increased biomarkers of inflammation (5-9).
**Restricting meat/ animal products**	Large intake of ω6-polyunsaturated fatty acids (PUFA), saturated and trans-fatty acids have pro-inflammatory and aggravating effects for SLE symptoms (10).<br>Moderate protein intake is associated with better immune function and delay in autoimmunity (10, 11).
**Increasing intake of vegetables, fruit, legumes and whole-grains**	Fibre, vitamins, minerals, isoflavones, phytochemicals, PUFA and other plant metabolites have anti-inflammatory effects. Many of these components positively diversify gut microbiota and mediate metabolic, inflammatory and immunity pathways (12).<br><br>High dietary fibre improves the synthesis of short chain fatty acids (SCFAs) in the metabolome and decreases the level of harmful free-radicals involved in disease state (13, 14).<br><br>High intake of ω3 PUFA reduces levels of pro-inflammatory cytokines such as IL-1, IL-6 and TNF (15). ω3 PUFA are also essential for the synthesis of eicosanoids, regulators of the inflammatory cascade (13).<br><br>Polyphenols from plants such as flavonoids have anti-inflammatory and antioxidant activity (16).
**Mood improvement**	Plant metabolites such as phytochemicals boost mood through their serotonergic, noradrenergic and dopaminergic effects (17).<br>ω3 PUFA modulate serotonin receptors in the cortex and hippocampus and increase brain-derived neurotrophic factor (BDNF) expression (17).<br>Vitamin C and magnesium antagonise the activity on N-methyl-D-aspartate (NMDA) receptors and increase BDNF (18, 19).<br><br>Decrease in pro-inflammatory molecules that negatively impact the circuitry in depression-related brain regions, such as the anterior cingulate cortex, amygdala and insula (20-22).

Supplemental Table 2. The literature identifies the main mechanisms through which WFPB diets could contribute to decreased inflammation and reduction in symptoms in SLE patients.
[1]List not exhaustive

Supplemental references:

1. Najjar R, Feresin R. Plant-Based Diets in the Reduction of Body Fat: Physiological Effects and Biochemical Insights. Nutrients. 2019;11(11):2712.

2. Francisco V, Pino J, Gonzalez - Gay MA, Mera A, Lago F, Gómez R, et al. Adipokines and inflammation: is it a question of weight? 2018. p. 1569-79.

3. Lerner A, Matthias T. Changes in intestinal tight junction permeability associated with industrial food additives explain the rising incidence of autoimmune disease. Autoimmunity reviews. 2015;14(6):479.

4. Dechristopher LR, Uribarri J, Tucker KL. Intake of high-fructose corn syrup sweetened soft drinks, fruit drinks and apple juice is associated with prevalent arthritis in US adults, aged 20–30 years. Nutrition & Diabetes. 2016;6(3):e199.

5. Hall KD, Ayuketah A, Brychta R, Cai H, Cassimatis T, Chen KY, et al. Ultra-Processed Diets Cause Excess Calorie Intake and Weight Gain: An Inpatient Randomized Controlled Trial of Ad Libitum Food Intake. Cell Metabolism. 2019;30(1):226-.

6. Aline Ester Da Silva Cruz L, Larissa Fortunato A, Renata Bertazzi L, Sandhi Maria B, Luana G. Association between consumption of ultra-processed foods and serum C-reactive protein levels: cross-sectional results from the ELSA-Brasil study. São Paulo Medical Journal. 2019;137(2):169-76.

7. Poti J, Braga B, Qin B. Ultra-processed Food Intake and Obesity: What Really Matters for Health—Processing or Nutrient Content? Curr Obes Rep. 2017;6(4):420-31.

8. Della Corte KW, Perrar I, Penczynski KJ, Schwingshackl L, Herder C, Buyken AE. Effect of Dietary Sugar Intake on Biomarkers of Subclinical Inflammation: A Systematic Review and Meta-Analysis of Intervention Studies. Nutrients. 2018;10(5).

9. Nicklas JM, Sacks FM, Smith SR, LeBoff MS, Rood JC, Bray GA, et al. Effect of Dietary Composition of Weight Loss Diets on High Sensitivity C-Reactive Protein: The Randomized POUNDS LOST Trial. Obesity (Silver Spring, Md). 2012;21(4).

10. Klack K, Bonfa E, Neto E. Diet and nutritional aspects in systemic lupus erythematosus. Revista Brasileira De Reumatologia. 2012;52(3):384-408.

11. Brown AB. Lupus Erythematosus And Nutrition. Journal of the American Dietetic Association. 1995;95(9):A31-A.

12. Sakkas H, Bozidis P, Touzios C, Kolios D, Athanasiou G, Athanasopoulou E, et al. Nutritional Status and the Influence of the Vegan Diet on the Gut Microbiota and Human Health. Medicina. 2020;56(2).

13. De Angelis M, Garruti G, Minervini F, Bonfrate L, Portincasa P, Gobbetti M. The Food-gut Human Axis: The Effects of Diet on Gut Microbiota and Metabolome. Current Medicinal Chemistry. 2019;26(19):3567-83.

14. De Filippis F, Pellegrini N, Vannini L, Jeffery IB, La Storia A, Laghi L, et al. High-level adherence to a Mediterranean diet beneficially impacts the gut microbiota and associated metabolome. Gut. 2015;65(11).

15. Breymeyer KL, Lampe JW, McGregor BA, Neuhouser ML. Subjective mood and energy levels of healthy weight and overweight/obese healthy adults on high-and low-glycemic load experimental diets. Appetite. 2016;107:253-9.

16. Joseph SV, Edirisinghe I, Burton-Freeman BM. Fruit Polyphenols: A Review of Anti-inflammatory Effects in Humans. Critical reviews in food science and nutrition. 2015;56(3):419-44.

17. Bahramsoltani R, Farzaei MH, Farahani MS, Rahimi R. Phytochemical constituents as future antidepressants: a comprehensive review. Reviews in the Neurosciences. 2015;26(6):699-719.

18. Eby GA, Eby KL. Rapid recovery from major depression using magnesium treatment. Medical Hypotheses. 2006;67(2):362-70.
19. Vines A, Delattre AM, Lima MMS, Rodrigues LS, Suchecki D, Machado RB, et al. The role of 5-HT1A receptors in fish oil-mediated increased BDNF expression in the rat hippocampus and cortex: A possible antidepressant mechanism. Neuropharmacology. 2012;62(1):184-91.
20. Felger JC. Imaging the Role of Inflammation in Mood and Anxiety-related Disorders. Current Neuropharmacology. 2018;16(5):533-58.
21. Cheatham RA, Roberts SB, Das SK, Gilhooly CH, Golden JK, Hyatt R, et al. Long-term effects of provided low and high glycemic load low energy diets on mood and cognition. Physiology & Behavior. 2009;98(3):374-9.
22. Harrison NA, Brydon L, Walker C, Gray MA, Steptoe A, Critchley HD. Inflammation Causes Mood Changes Through Alterations in Subgenual Cingulate Activity and Mesolimbic Connectivity. Biological Psychiatry , 66 (5) 407 - 414 (2009). 2009.
